# Comparison of stool antigen test with gastric biopsy for the detection of Helicobacter Pylori infection

**DOI:** 10.12669/pjms.291.2865

**Published:** 2013

**Authors:** Majid Sharbatdaran, Mehrdad Kashifard, Shahriar Shefaee, Sepideh Siadati, Babak Jahed, Samaneh Asgari

**Affiliations:** 1 Majid Sharbatdaran, Assistant Professor, Department of Pathology, Babol university of Medical Sciences, Babol, Iran.; 2 Mehrdad Kashifard, Associate Professor, Department of Internal Medicine, Babol university of Medical Sciences, Babol, Iran.; 3 Shahriar Shefaee, Assistant Professor, Department of Pathology, Babol university of Medical Sciences, Babol, Iran.; 4 Sepideh Siadati, Assistant Professor, Department of Pathology, Babol university of Medical Sciences, Babol, Iran.; 5 Babak Jahed, Pathologist, Babol university of Medical Sciences, Babol, Iran.; 6 Samaneh Asgari, Master of Biostatistics, Clinical Research Development Center, Shahid Beheshti Hospital, Babol, Iran.

**Keywords:** Gastric biopsy, Helicobacter Pylori stool antigen, Dyspepsia, Sensitivity, Specificity, Negative predictive value

## Abstract

***Objective: ***Helicobacter Pylori (H.pylori) is one of the most important causes of dyspepsia and diagnosis can be made by invasive or non-invasive methods. One of the non-invasive methods, H.pylori stool antigen test (HpSA) is simple, fast and relatively inexpensive. According to this view with regard to gastric biopsy as a gold standard the sensitivity, specificity, positive and negative predictive values of this method were calculated.

***Methodology:*** Stool samples of 61 patients who underwent upper endoscopy and gastric biopsy due to dyspepsia were evaluated for H. Pylori stool antigen using sandwich ELISA method.

***Results:*** From the 61 patients who participated in this study, H.pylori was diagnosed in 38 (62.3%) gastric biopsies, 25(66%) of these had positive HpSA test. Also, of 27 (37.7%) positive HpSA cases, H.pylori was seen in 25 gastric biopsies. For this method, sensitivity of 66% with 93% positive predictive value was calculated. Also, 91% specificity with 62% negative predictive value was estimated.

***Conclusion:*** High positive HpSA indicates high risk of H.pylori infection and high specificity shows that the likelihood of false positive is low. Therefore, physicians can trust on this method and start patient`s treatment.

## Introduction

 Diagnostic methods for detection of Helicobacter pylori (H. pylori) infection are divided into two types: invasive and non-invasive. The invasive methods are the following: biopsy and histology, rapid urease testing and culture require endoscopy. They are expensive and difficult to perform in young children.^1^^-^^3^ The non-invasive methods have recently been increased. One of them, 13C- urea breath test despite high sensivity and specificity has a relatively high cost and requires trained staff and well equipped laboratory instruments. Also, the low specificity and complicated performance in children are the other limitations of this test.^1^^-^^6^ The serological tests are the other non-invasive diagnostic methods.

 Since antibodies may remain after eradication especially in children, it lacks reliability and is not an appropriate method for post-treatment follow up.^1^^,^^2^ H.pylori stool antigen test (HpSA) is another non-invasive method using a commercial kit based on enzyme – linked immunosorbent assay (ELISA) technique by means of monoclonal or polyclonal antibodies against H.pylori. This technique has several advantages over the other techniques. The sensitivity (94%) and specificity (81%) are high and is used for diagnosis as well as monitoring after treatment. It is not only simple, fast and inexpensive but also requires no laboratory attendance (only stool sample is needed) or fasting which is required for endoscopy and urea breath test. Therefore, this method is particularly useful in children.^1^^-^^3^^,^^6^ The new generated monoclonal HpSA test replace the less reliable polyclonal antibodies.

 The objective of the present study was to evaluate the diagnostic value of HpSA using the current kit in Iran in comparison to biopsy and histology as a gold standared method. Then, sensitivity, specificity, positive and negative predictive values were calculated.

## Methodology

 This study was done on 61 consecutive outpatients under 45 years of age with dyspeptic symptoms. All the patients provided written consent and this study which was approved by the Ethics committee of Babol University of Medical Sciences. The exclusion criteria were the presence of gastrointestinal bleeding during endoscopy, gastric carcinoma and treatment with antibiotic, Bismuth or proton-pump inhibitor four weeks prior to collecting stool samples. Upper endoscopy was performed in all patients and one biopsy specimen of the antrum and one of the corpus were obtained from each patient. The specimens were fixed in formalin, stained with hematoxylin and eosin and modified Giemsa and examined for the presence of H.pylori by two independent pathologists. They were unaware of the results of the HpSA test. H.pylori positive status was defined if H.pylori was identified in histologic examination.

 A fresh stool sample was collected from each patient and stored at -20c until analyzed. HpSA test (GA Generic Assay, Germany) was performed according to manufacturer recommendation. Those performing and reading the test had no knowledge of the H.pylori status of the patients. The HpSA test is a qualitative, sandwich ELISA method using polyclonal H.pylori antibodies adsorbed to microwells as capture antibody. After stool samples were unfrozen, small portion of each sample emulsified with diluent by using an applicator stick. 100 µl of diluted stool samples and negative and positive controls were transferred to the wells. After incubation at room temperature and thorough washing, rabbit polyclonal anti-H.pylori antibody conjugated with Biotin was added to each well.

 Again 30 minutes incubation at room temperature and thorough washing was done. Then, Avidin conjugated with horseradish peroxidase (HRP) was dispensed to each well, incubated at room temperature for 30 minutes and then tetramethybenzidine (TMB) and H2O2 was added to each well. After 15 minutes incubation at dark, 120 µl of a stopping solution (sulfuric acid) was added and the optical density (OD) for each well was obtained at 450 nm and 630 nm as reference wavelengths by spectrophotometer (Avernest, stat fax 3200, USA). According to the manufacturer instruction, the cut-off value was obtained by the mean OD of negative control at 450nm, plus 0.1. OD ≤ cut-off was defined negative, OD> cut-off was considered positive.

## Results

 Sixty one patients (25 men and 36 women) with mean age of 31.1±7.5 years complaining of dyspeptic symptoms were enrolled in this study. H. pylori was diagnosed in 38(62.3%) gastric biopsies and 25 of these had positive HpSA. Also, of 27 (37.7%) stool samples with positive HpSA test, H.pylori was positive in 25 gastric biopsies. The statistical results are shown in Table-I.

## Discussion

 The most important finding of this study was high specificity (91.3%) of the HpSA test pointing to low rate of false positive. Also, high positive predictive value (92.6%) indicates the high probability of H. pylori infection. The first experience of HpSA method in Iran was published in 2003. Their study was conducted on 54 patients with gastrointestinal problems. The sensitivity of HpSA before treatment was 78.6% (higher than our study, 65.8%), specificity was 92.3% (nearly equal to our finding, 91.3%).^7^ In 2005 a study was carried out on 100 children with dyspeptic symptoms in Tabriz, Iran. They compared three diagnostic methods (histology, serological test and HpSA). HpSA sensitivity and specificity was 54.8% and 79.4%, respectively.^8^

**Table-I:** The statistical results of H. pylori stool antigen test

		*95% Concidence Intervals*
Sensitivity	66%	51 to 81
Specificity	91%	80 to 100
Positive predictive value	93%	83 to 100
Negative predictive value	62%	45 to 78
Likelihood Ratio+	7.57	1.97 to 29.0
Likelihood Ratio -	0.37	0.24 to 0.59

 All comparative studies which were done outside IRAN since 2000, showed different results.^9^^-^^17^ User manual of this commercial kit specifies sensitivity of 91% and specificity of 100%. For explanation of these obvious differences, the following reasons are most likely to be considered:

1. These commercial kits are manufactured abroad, therefore storage and transportation can affect the quality of these kits (the best storage temperature is – 2 to – 8 c). On the other hand, HpSA kits typically expire at a maximum of two months after the date of manufacture. Considerable transport time can influence performance of the test.
2. According to the instruction, this commercial kit is able to detect 15ng/ml of H. pylori antigen. There may be a decrease in gastric bacterial load of the patients due to overconsumption of antibiotics in Iran that plays an inhibitory role on bacterial growth.
3. Quality control and human error in laboratory performance could also justify the difference.
4. There are a number of technical errors that can affect HpSA test result. Some of these problems are presented as:

- Cross contamination that can lead to false positive.
- Incorrect dilution.
- Insufficient homogenization after dilution.
- Fermented samples with PH values below 5 after suspension preparation may produce false negative results.
- The instruction cut-off is according to company protocol. A new cut- off may increase performance effectiveness.

The present study shows a very high specificity (91.3%) of the HpSA test that means a positive result from the HpSA test is low in patients without H.pylori infection. (low number of false positive about 1 in 10 tests). Also, high positive predicative value (92.6%) indicates that if HpSA test is positive, the probability of H. pylori infection should be considered.

 It can be concluded that the positive HpSA test has a good sensitivity for the diagnosis of H. pylori infection but negative HpSA test is not reliable enough to rule out H.pylori infection. Therefore, with respect to the results of other studies and also this study, HpSA test can be a reliable method for detection of H.pylori infection and physicians have to be more careful in their interpretation. Test and treat approach was recommended at Maastricht III consensus for patients with dyspeptic symptoms referred to primary level of medical services. This method can be applied in patients below 45 years old, without reflux symptoms, no NSAID consumption and no high risk symptoms (like weight loss). As a consequence, the HpSA test for the detection of H.pylori infection seems to be a good alternative for invasive diagnostic tests such as urea breath test, especially in our country.
